# Supplementary material for: Oral immune dysfunction is associated with the expansion of FOXP3+PD-1+Amphiregulin+ T cells during HIV infection
Source: Nat Commun. 2021 Aug 26;12:5143. doi: 10.1038/s41467-021-25340-w (PMC8390677; doi:10.1038/s41467-021-25340-w)
Supplement: Supplementary file 1 — Supplementary Information [file 41467_2021_25340_MOESM1_ESM.pdf]

Supplementary Figures and Table

Oral Immune Dysfunction is Associated with the Expansion of FOXP3^{+}PD-1^{+}Amphiregulin^{+} T Cells during HIV infection

Supplementary Fig. 1

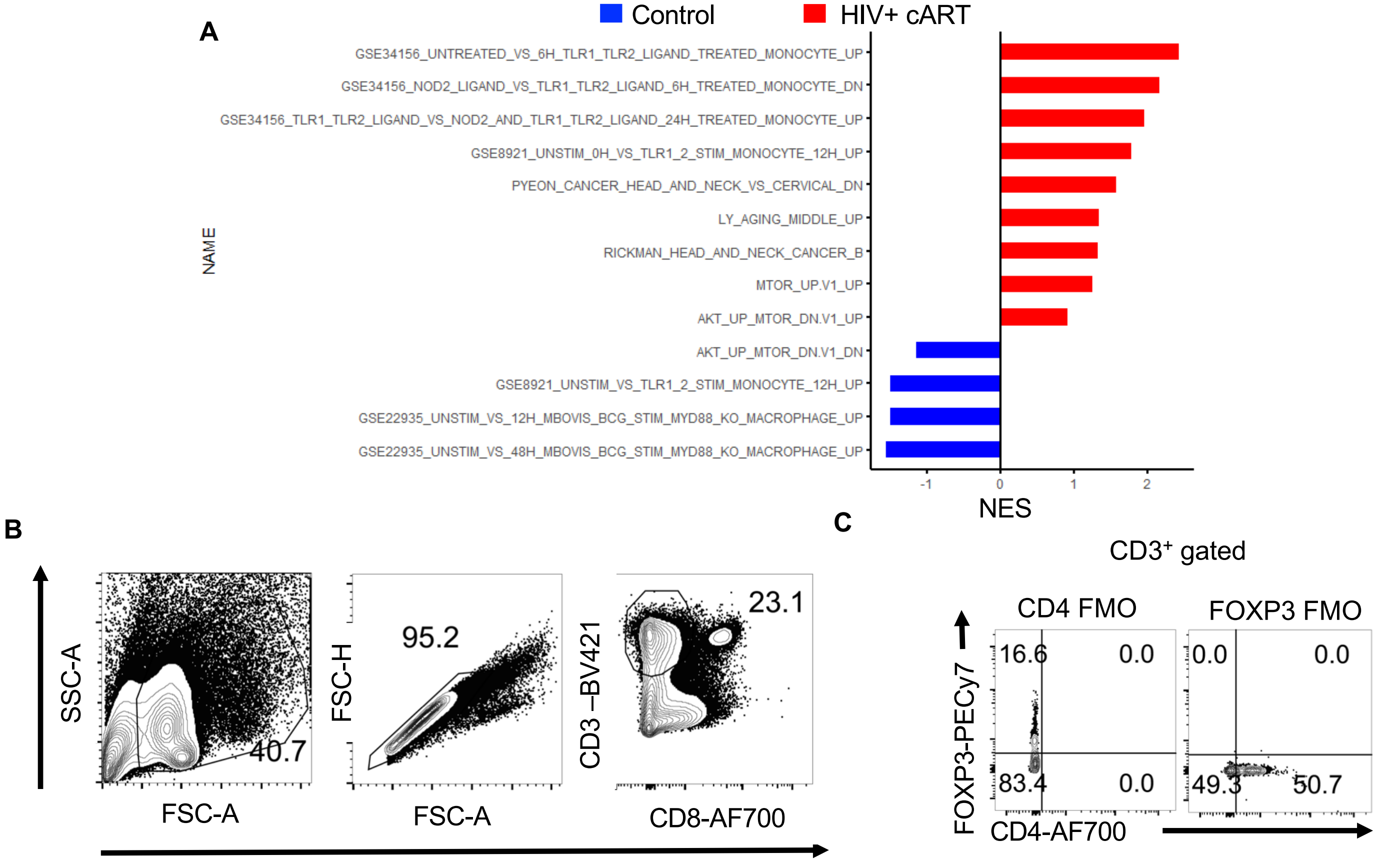

Supplementary Fig. 1. Oral Mucosa characterization. A) Gingival cells were enriched for immune cells by reducing the epithelial cells through gradient centrifugation before transcriptome analyses (n=3 HIV+cART; n=3 uninfected healthy controls). Normalized Enrichment Score (NES) showing the enrichment of pathways of aging, head and cancer and Akt signaling in oral mucosa of HIV^{+} patients based on gene sets in MSigDB. (B) Gating strategy used for the flow cytometry analysis exemplifying the gates in figures 1E, 2D, 8A-D, and supplementary figures 2, 4, and 19. HOIL were processed *ex vivo* for flow cytometry. CD4^{+} T cells were gated by either gating on CD8 negative CD3^{+} cells, or CD4^{+}CD3^{+} cells. CD8-negative gating approach (B), and FMO controls for determining the positive boundaries for CD4 and FOXP3 staining (C) are shown. These FSC/SCC, singlet, and T cell/T_{reg} gating were used for all the *ex vivo* analysis of oral mucosa. Source data are provided as a Source Data file.

Supplementary Fig. 2

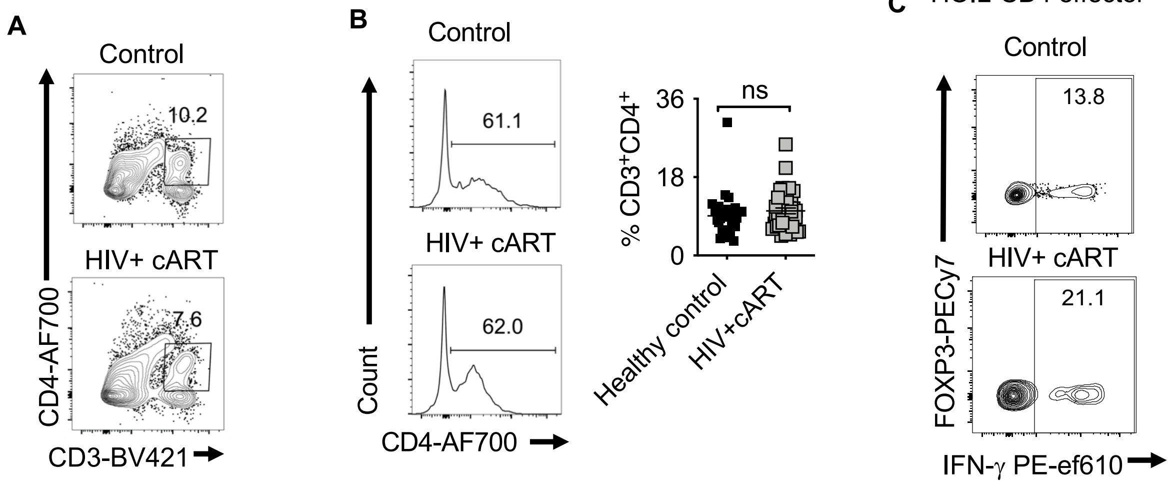

Supplementary Fig. 2. Oral Mucosa characterization. HOIL from study participants were processed *ex vivo*. CD4 cell gating (A), histogram plots (B, left), statistical analyses for CD4 expression in CD3 population (B, right), and IFN-γ expression in $CD4^+$ FOXP3 negative T cells (C). B) n=32 uninfected healthy controls; n=46 HIV+ cART; Mean values ± SEM are plotted. Mann Whitney test was performed and the differences were non-significant (ns).

Supplementary Fig. 3

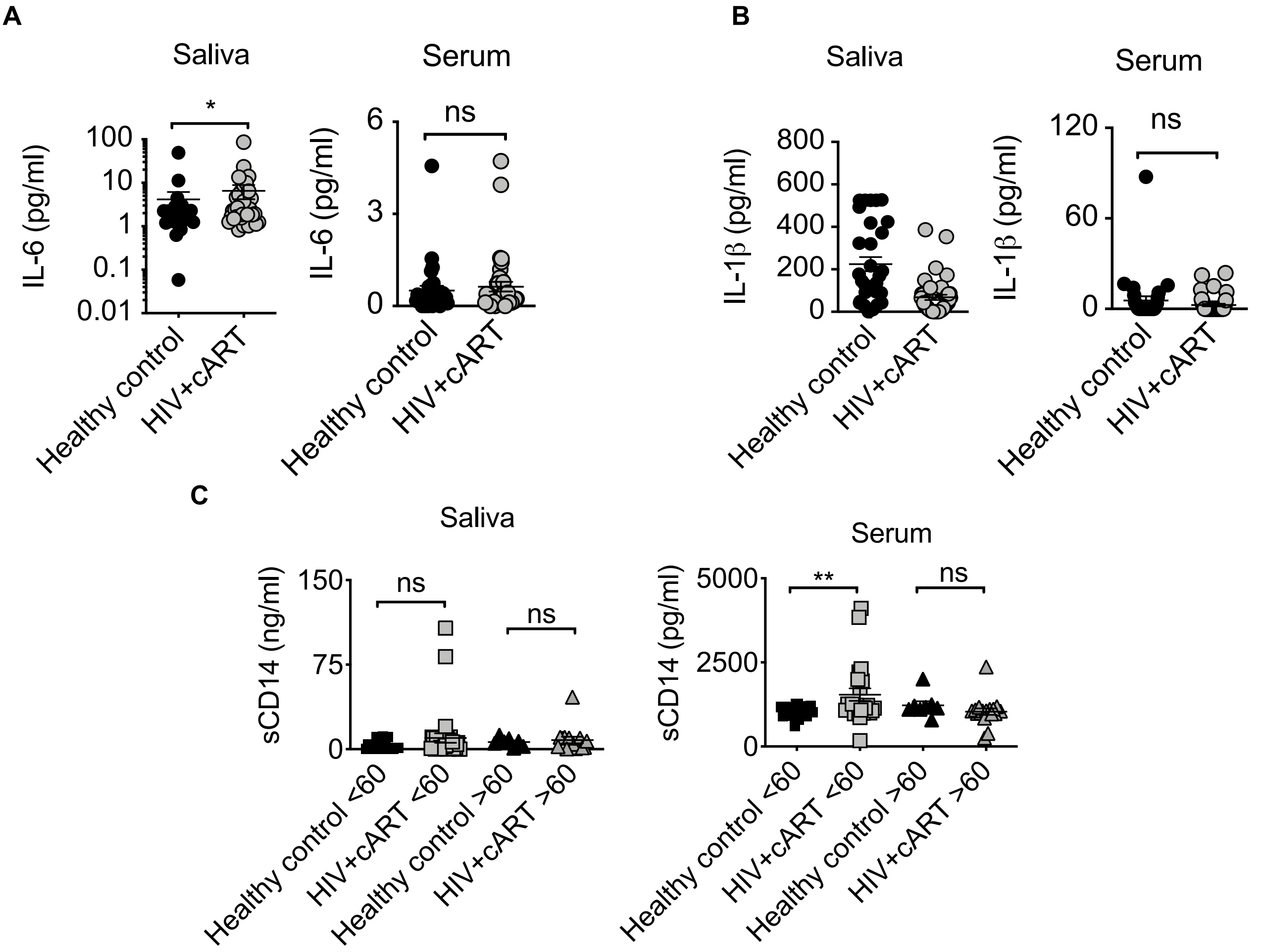

Supplementary Fig. 3. Comparison of saliva and serum of HIV+ cART patients. Saliva and serum from study participants (n=46 HIV+ cART; n=32 uninfected healthy controls) were processed for ELISA. ELISA quantification of IL-6 (A) and IL-1β (B) and soluble CD14 (C) in saliva and serum. A-C) Mean values ± SEM; * $P<0.05$ Two-tailed; Mann Whitney test. Source data are provided as a Source Data file.

Supplementary Fig. 4

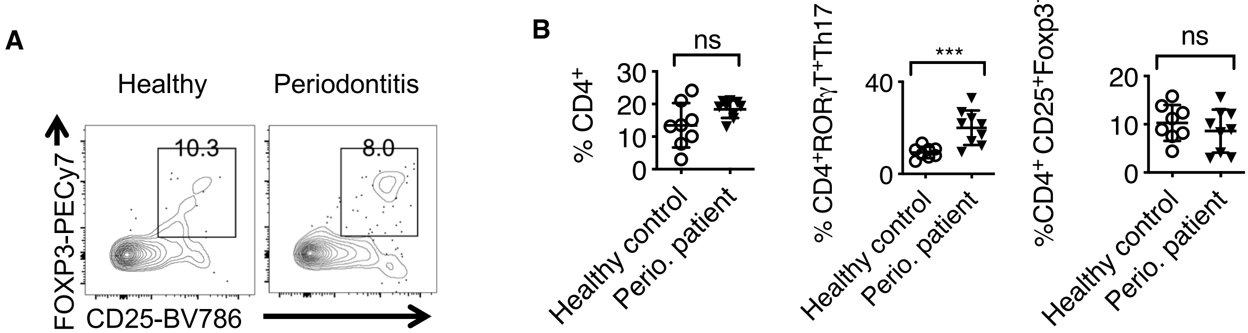

Supplementary Fig. 4. **No T_{reg} alterations were observed in the oral mucosa of periodontitis patients *ex vivo*.** HOILs from gingival mucosa were collected under a separate approved IRB protocol-University Hospitals Cleveland Medical Center Institutional Review Board (UH-IRB number: 03-13-15). They were processed for flow cytometry. **A**) CD25 and FOXP3 expression in $CD3^{+}CD4^{+}$ gated HOIL cells **B**) Statistical analyses comparing % $CD4^{+}$ T cells, Th17 cells, and T_{regs}, comparing the two groups. Healthy control (n=8; 5 females and 3 males) and periodontitis patients (Perio. n= 9; 6 females and 3 males); Mean values ± SEM; * P< 0.05 Two-tailed ; Mann Whitney test.

Supplementary Fig. 5

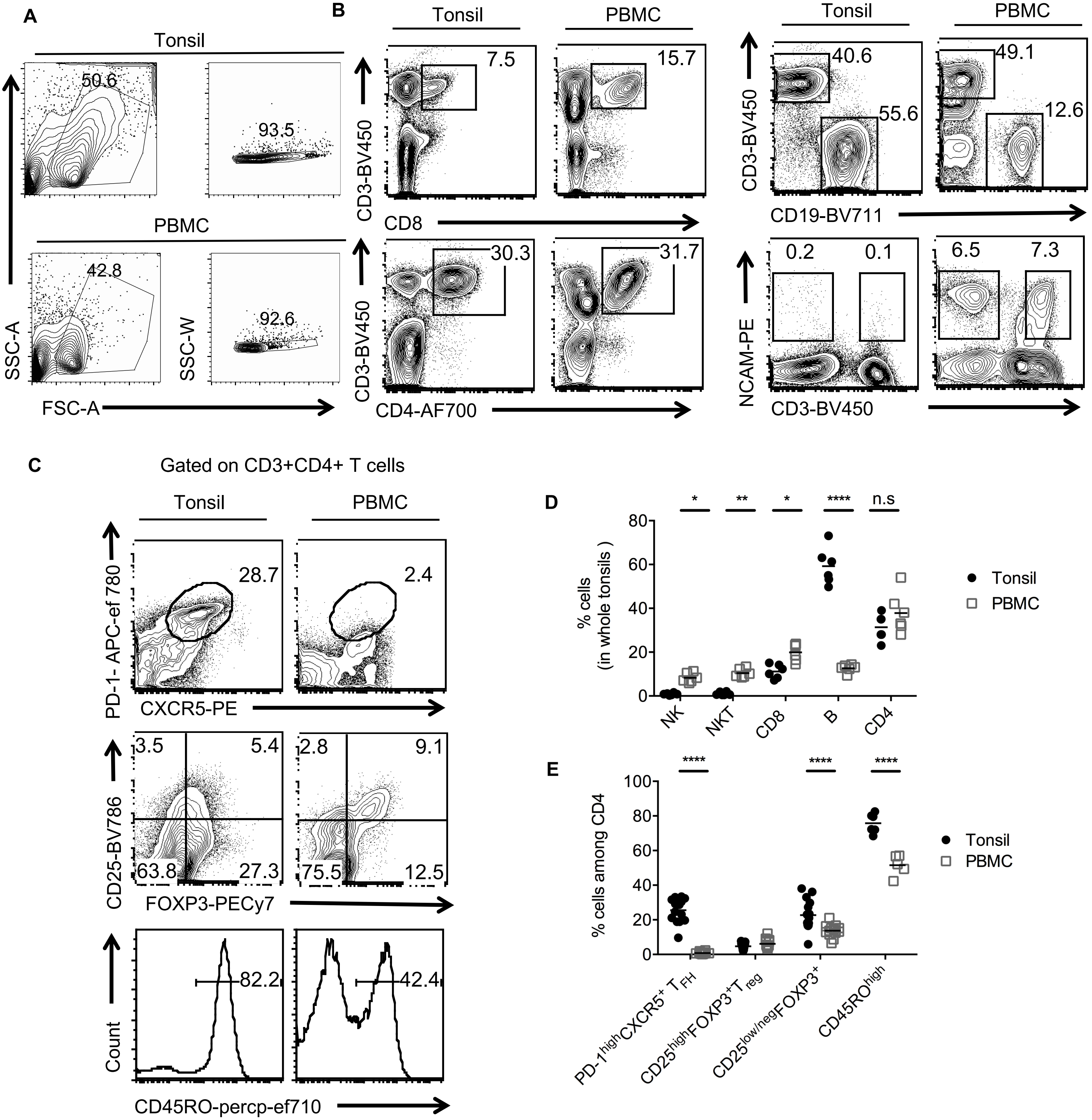

Supplementary Fig. 5. Gating strategy used in tonsil staining and characterization of immune cells in tonsils in comparison with PBMC cells *ex vivo*. Tonsils and PBMC were processed *ex vivo*. FSC/SSC, singlet gates (A) and subsequent CD4 gating (gated on CD3+CD8 negative cells) (B, left), exemplifying the gates used in figures 3, 4, 5, 6, 7 and supplementary figures 6-18. Flow cytometry plots showing NK and B cells (B, right). C) CXCR5 and PD-1 (top), CD25, FOXP3 (middle) and CD45RO expression in $CD4^{+}$ T cells. Additional gating strategies used for FOXP3+ and PD-1+ cells tonsillar cultures (cells were pre-gated as in A,B). D,E). Statistical analyses and comparison between PBMC and tonsils for the indicated populations (from at least 5 independent donors). D,E) Each data point represents a donor; Mean values ± SEM; * P< 0.05 Two-tailed; Mann Whitney test.

Supplementary Fig. 6

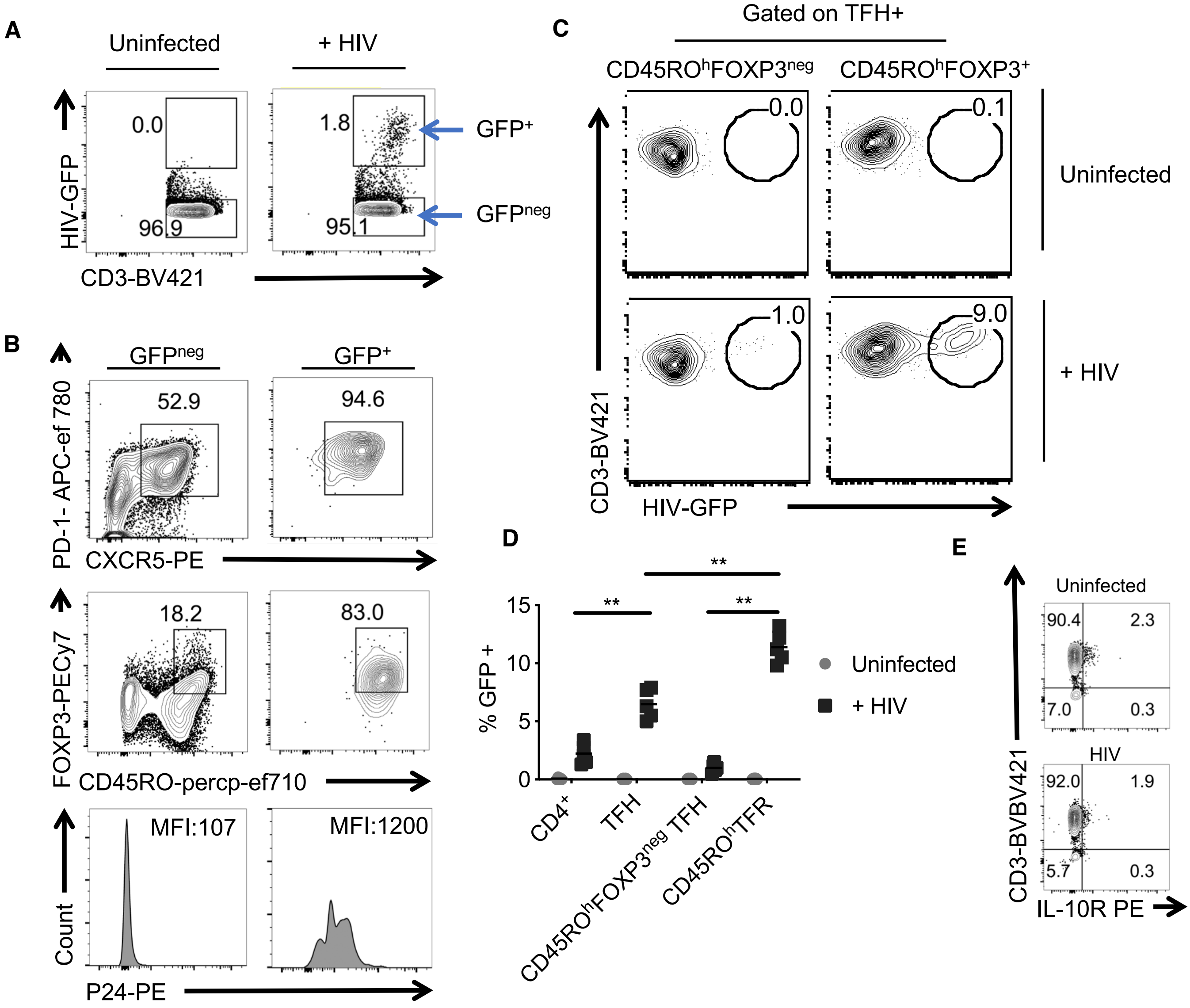

Supplementary Fig. 6. Characterization of HIV- infected cells in tonsils. A) Whole human tonsil cultures (HTC) were activated by TCR stimulation and allowed to expand in the presence of TGF-β1 (10 ng/ml) and IL-2 (100 U/ml). They were infected with HIV on day 2 after TCR stimulation. Productive infection, as determined by GFP expression 72 hours post-infection in HTC. B) PD-1 and CXCR5 expressing TFH (top), CD45RO, FOXP3 (middle) and p24 expression in GFP negative and GFP^+ $CD4^+$ cells. C) GFP expression in $CD45RO^{high}FOXP3^{neg}$ and $CD45RO^{high}FOXP3^+$ cells in TFH population. D) Statistical analyses of % GFP+ cells in indicated population. Representative flow cytometric data and statistical analyses from 5 independent tonsil donors are shown. Mean values ± SEM; * $P< 0.05$ Two-tailed; Mann Whitney test. E) **IL-10 receptor was unaltered in $CD4^+$ cells in HIV-infected cultures** . Purified $CD4^+$ cells were activated and infected with HIV as above. Flow cytometry was performed to determine IL-10 receptor expression in $CD4^+$ T cells, 6 days post-infection. Representative flow cytometric data from three independent tonsil donors.

Supplementary Fig. 7

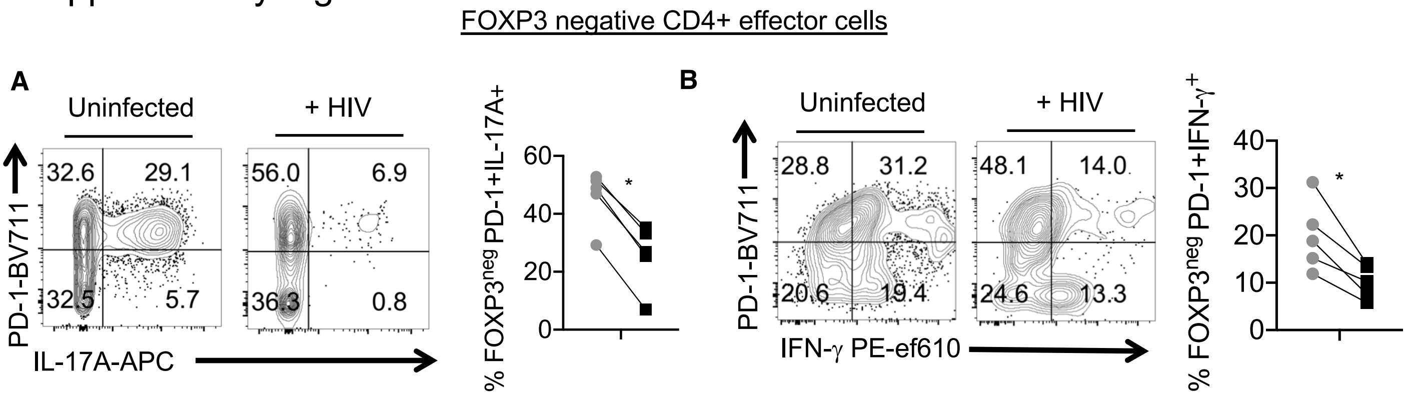

Supplementary Fig. 7. HIV infection resulted in the reduction of cytokine expressing CD4^{+} T cell effectors in tonsils. HTC were activated by TCR stimulation and infected as in Fig.3. PD-1 and IL-17A expression (A), and PD-1 and IFN-γ expression (B) in FOXP3 negative CD4^{+} effector cells 5 days post-infection. A,B) n = 5; * $P< 0.05$; Two-tailed; Mann Whitney test.

Supplementary Fig. 8

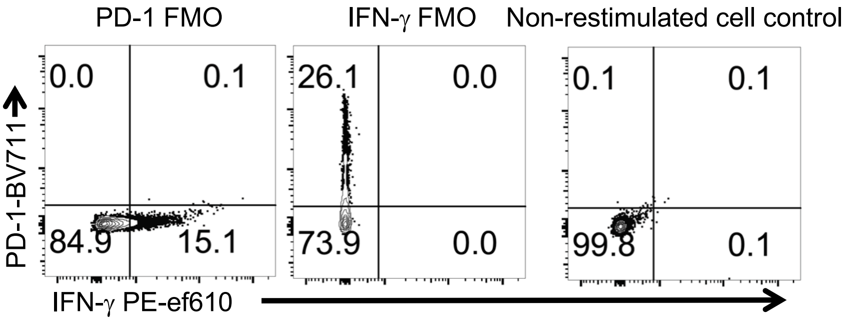

Supplementary Fig. 8. FMO controls to determine the boundary for positively stained cells. HTC were activated by TCR stimulation and allowed to expand in the presence of TGF-β1 (10 ng/ml) and IL-2 (100 U/ml). They were infected with HIV on day 2 after TCR stimulation. PD-1, IFN-γ FMO controls, and non-restimulated cell control for the IFN-γ and PD-1 staining (gated on $CD4^+FOXP3^+$ cells), 5 days post-infection.

Supplementary Fig. 9

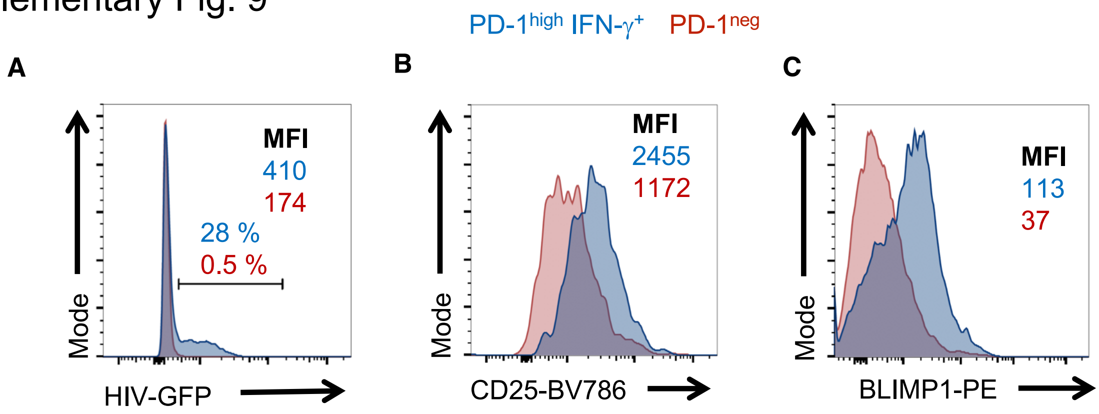

Supplementary Fig. 9. Characterization of PD-1^{high}IFN-γ^{+} PD-1^{low} cells in HIV- infected tonsils. HTC were activated by TCR stimulation and allowed to expand in the presence of TGF-β1 (10 ng/ml) and IL-2 (100 U/ml). They were infected with HIV on day 2 after TCR stimulation. GFP (A), CD25(B), and BLIMP-1(C) expression in CD4^{+}FOXP3^{+} cells 6 days post-infection. Representative flow cytometric data from 3 independent tonsil donors are shown.

Supplementary Fig. 10

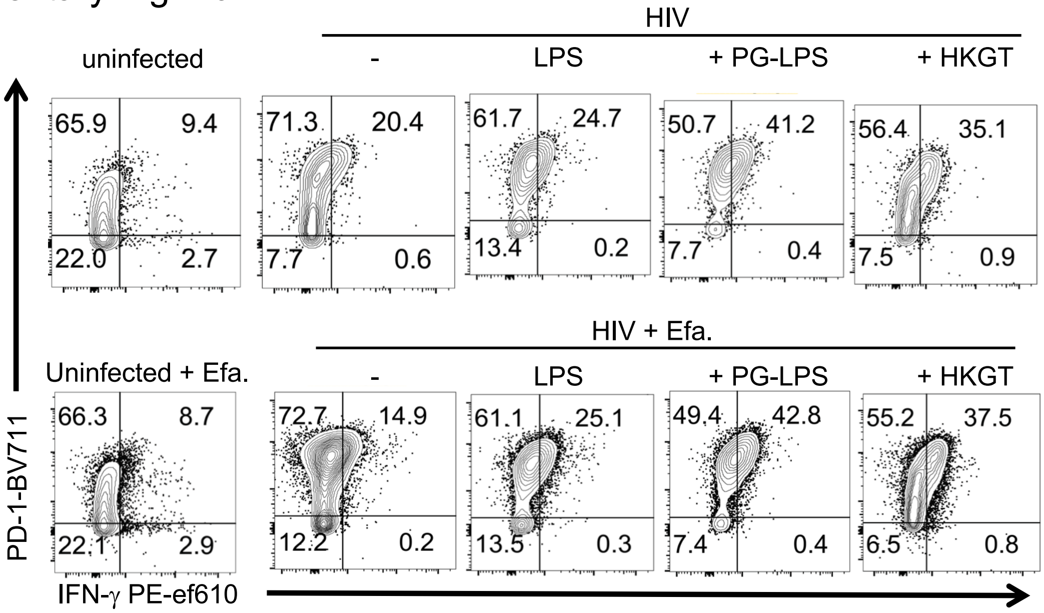

Supplementary Fig. 10. PD-1^{hi}IFN-γ^+ FOXP3$^+$ cell accumulation is enhanced by TLR-2 ligands in the context of HIV infection. A) Purified CD4$^+$ T cells were activated with TCR stimulation and allowed to expand in the presence of TGF-β1 (10 ng/ml) and IL-2 (100 U/ml). Some cultures were infected with HIV on day 2 after TCR stimulation. Indicated cytokines or reagents were also added during this time. PD-1 and IFN-γ expression in CD4$^+$FOXP3$^+$ cells 6 days post-infection. Representative flow cytometric data from 3 independent tonsil donors are shown.

Supplementary Fig. 11

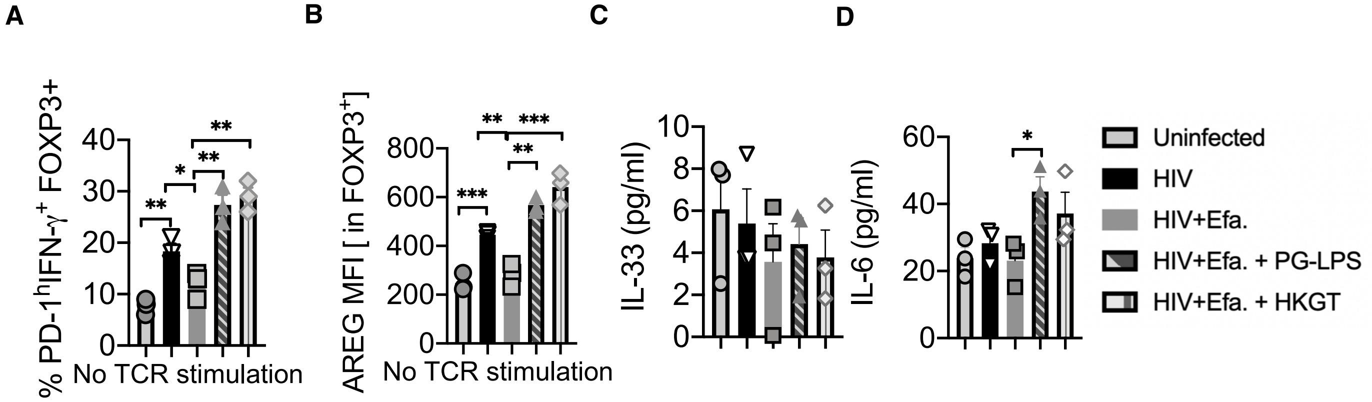

Supplementary Fig. 11. A,B) HIV induced PD-1^{h} IFN-γ^{+}FOXP3^{+} and AREG expression in FOXP3^{+} cells are independent of TCR stimulation. Purified and unstimulated CD4^{+} T cells were allowed to expand in the presence of TGF-β1 (10 ng/ml) and IL-2 (100 U/ml). Some cultures were infected with HIV on day 2 after TCR stimulation. Indicated cytokines or reagents were also added during this time. C,D) **Cytokine expression in tonsil CD4^{+} T cells during HIV infection.** Purified CD4^{+} T cells activated by TCR stimulation and infected as above. IL-33 (C) and IL-6 (D) were quantified by ELISA 3 days post-infection. A-D), Mean values +/- SEM are shown. * P< 0.05; ** <0.005, *** < 0.0005, as determined by unpaired t tests using the data from three independent experiments. Source data are provided as a Source Data file.

Supplementary Fig. 12

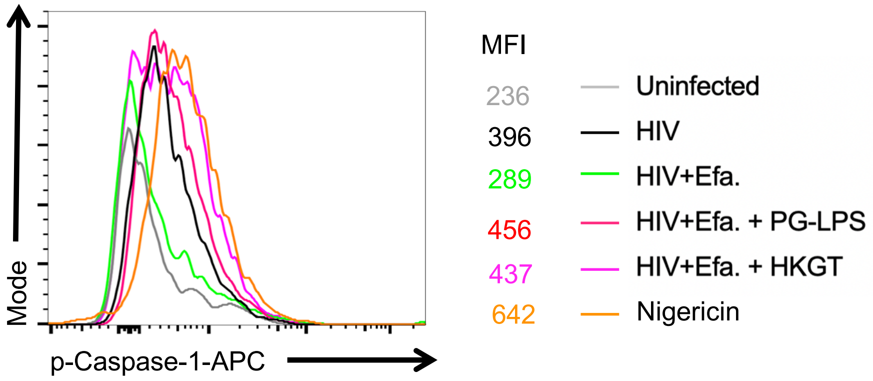

Supplementary Fig. 12. Activated caspase-1 expression is upregulated in tonsil $CD4^+$ T cells during HIV infection. Purified $CD4^+$ T cells were activated by TCR stimulation and allowed to expand in the presence of TGF-β1 (10 ng/ml) and IL-2 (100 U/ml). Some cultures were infected with HIV on day 2 after TCR stimulation. Indicated cytokines or reagents were also added during this time. Phosphorylated caspase-1 expression 3 days post-infection. Representative flow cytometric data from 3 independent tonsil donors.

Supplementary Fig. 13

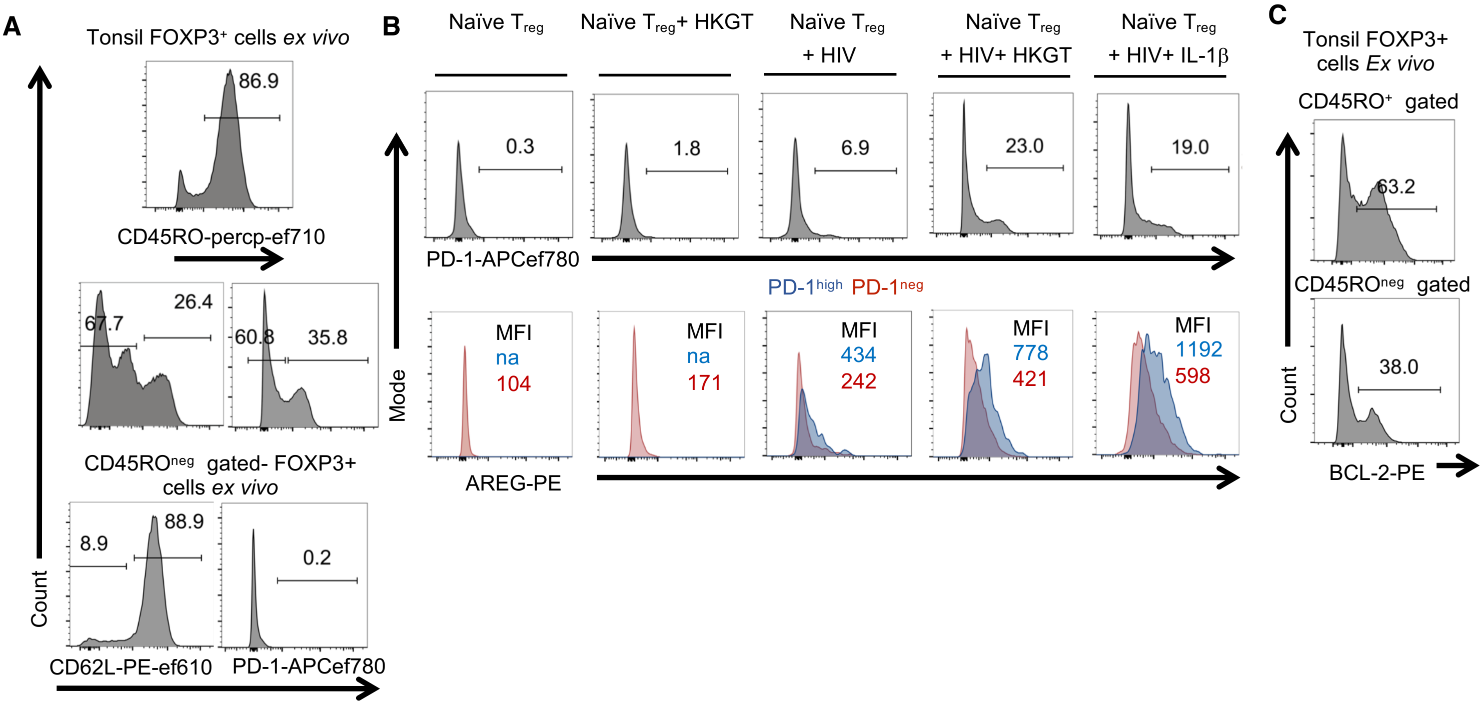

Supplementary Fig. 13. PD-1^{hi}IFN-γ^{+} FOXP3^{+} cell induction from naïve T$_{regs}$ is enhanced by TLR-2 ligands in the context of HIV infection. A, C) *Ex vivo* flow cytometry analysis of tonsils (gated on CD4^{+}FOXP3^{+} cells). B) CD4^{+}CD45RO^{neg}CD127^{low}CD25^{+} T$_{reg}$ cells were sorted using three-step sorting of the tonsil cells. First, EasySep™ Human CD4+ T Cell Isolation Kit (STEMCELL Technologies) was used to isolate untouched CD4+ T cells using depletion of non-CD4 T cells. Then human CD45RO^{+}CD4^{+} kit (Miltenyi biotech) was used to remove CD4 memory cells. Purified CD45RO^{neg} naïve (92%) were used for further purification of T$_{regs}$ using human CD4+CD127lowCD25+ regulatory T cell kit (STEMCELL Technologies) (> 85% FOXP3^{+}). These cells were activated with TCR stimulation and allowed to expand in the presence of TGF-β1 (10 ng/ml) and IL-2 (100 U/ml). Some cultures were infected with HIV on day 2 after TCR stimulation. Indicated cytokines or reagents were also added during this time. Flow cytometry was performed on day 7 after infection. Representative flow cytometric data from 3 independent tonsil donors are shown.

Supplementary Fig. 14

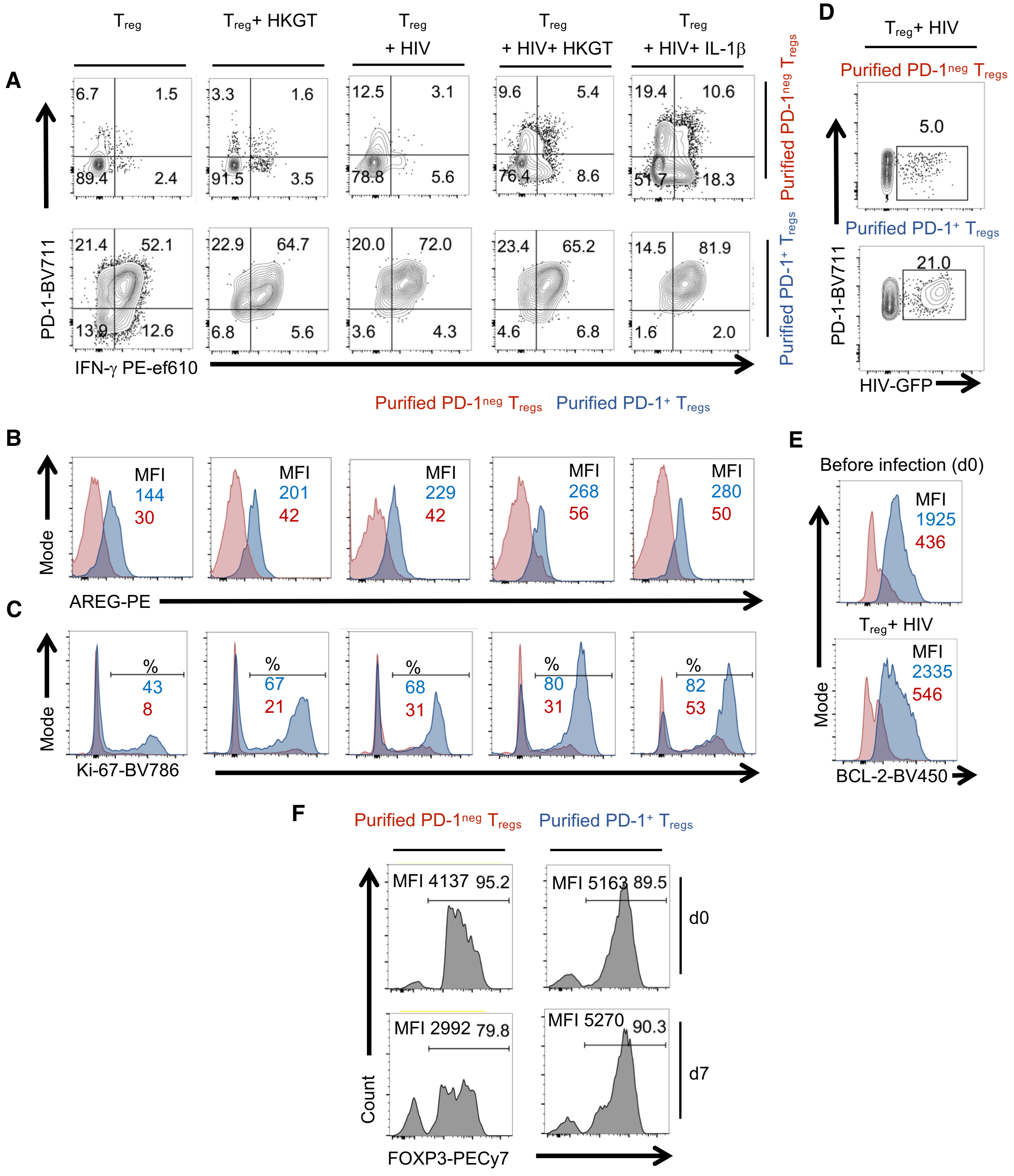

Supplementary Fig. 14. PD-1^{hi}IFN-γ^{+} FOXP3^{+} cell induction and proliferation are enhanced by TLR-2 ligands in the context of HIV infection. CD4^{+}PD-1^{neg}CD127^{low}CD25^{+} T$_{reg}$ and CD4^{+}PD-1^{+}CD127^{low}CD25^{+} T$_{reg}$ cells were sorted using three-step sorting of the tonsil cells. EasySep™ Human CD4+ T Cell Isolation Kit (STEMCELL Technologies) was used to isolate untouched CD4^{+} T cells using depletion of non-CD4 T cells. Anti-Biotin multisort kit (Miltenyi biotech) was used to positively sort anti-PD-1-biotin labelled cells. The beads were removed from positively sorted cells. Purified CD4^{+}PD-1^{+} and CD4^{+}PD-1^{neg} (~90-95%) were used for further purification of T$_{regs}$ using human CD4+CD127lowCD25+ regulatory T cell kit (STEMCELL Technologies) (> 85% FOXP3^{+}). These cells were activated with TCR stimulation and allowed to expand in the presence of TGF-β1 (10 ng/ml) and IL-2 (100 U/ml). Some cultures were infected with HIV on day 2 after TCR stimulation. Indicated cytokines or reagents were also added during this time. Flow cytometry was performed on day 7 after infection to determine PD-1, IFN-γ, (A), AREG (B), Ki-67 (C), and GFP (D). BCL-2 (E) and FOXP3 (F) expression on day 0 (d0) and d7 after HIV infection. Representative flow cytometric data from 3 independent tonsil donors are shown.

Supplementary Fig. 15

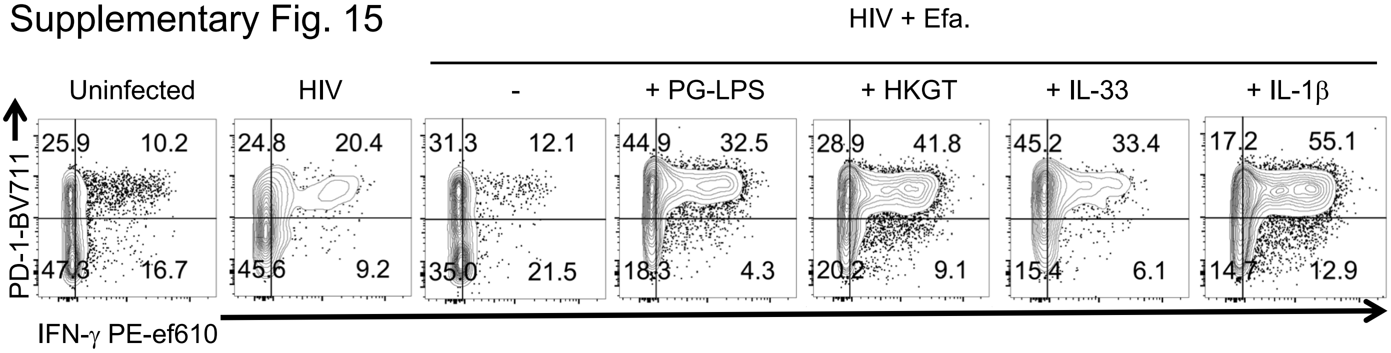

Supplementary Fig. 15. PD-1^{hi}IFN-γ^{+} FOXP3^{+} cell induction is enhanced by TLR-2 ligands, IL-33 and IL-1β in the context of HIV infection. Purified CD4^{+} T cells were activated by TCR stimulation and allowed to expand in the presence of TGF-β1 (10 ng/ml) and IL-2 (100 U/ml). Some cultures were infected with HIV on day 2 after TCR stimulation. Indicated cytokines or reagents were also added during this time. PD-1 and IFN-γ expression in CD4^{+}FOXP3^{+} cells 6 days post-infection.

Supplementary Fig. 16

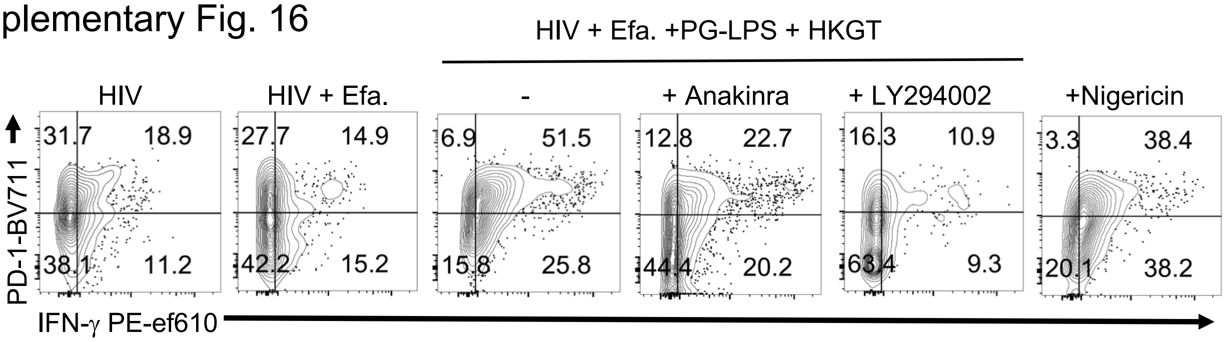

Supplementary Fig. 16. PD-1^{hi}IFN-γ^+ FOXP3$^+$ cell induction requires Akt-1 dependent IL-1β and inflammasome signaling in the context of HIV infection. Purified CD4$^+$ T cells were were activated by TCR stimulation and allowed to expand in the presence of TGF-β1 (10 ng/ml) and IL-2 (100 U/ml). Some cultures were infected with HIV on day 2 after TCR stimulation. Indicated cytokines or reagents were also added during this time. PD-1 and IFN-γ expression in CD4$^+$FOXP3$^+$ cells 6 days post-infection. Representative flow cytometric data from five independent tonsil donors are shown.

Supplementary Fig. 17

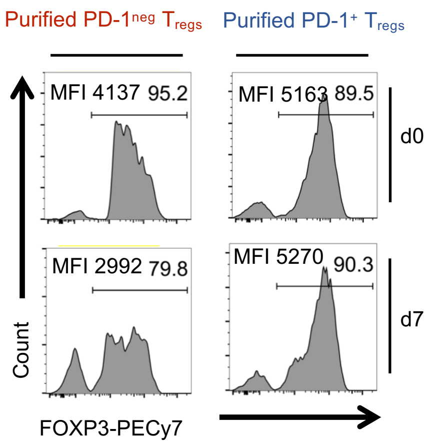

Supplementary Fig. 17. PD-1^{neg} FOXP3^{+} cells lose FOXP3 expression in the context of HIV infection. CD4^{+}PD-1^{neg}CD127^{low}CD25^{+} T$_{reg}$ and CD4^{+}PD-1^{+}CD127^{low}CD25^{+} T$_{reg}$ cells were sorted using three-step sorting of the tonsil cells. EasySep™ Human CD4+ T Cell Isolation Kit (STEMCELL Technologies) was used to isolate untouched CD4^{+} T cells using depletion of non-CD4 T cells. Anti-Biotin multisort kit (Miltenyi biotech) was used to positively sort anti-PD-1-biotin labeled cells. The beads were removed from positively sorted cells. Purified CD4^{+}PD-1^{+} and CD4^{+}PD-1^{neg} (~90-95%) were used for further purification of T$_{regs}$ using human CD4+CD127lowCD25+ regulatory T cell kit (STEMCELL Technologies) (> 85% FOXP3^{+}). These cells were activated with TCR stimulation and allowed to expand in the presence of TGF-β1 (10 ng/ml) and IL-2 (100 U/ml). Some cultures were infected with HIV on day 2 after TCR stimulation. Flow cytometry was performed to determine FOXP3 expression on day 0 (d0) and d7 after HIV infection. Representative data from 3 independent tonsil donors are shown.

Supplementary Fig. 18

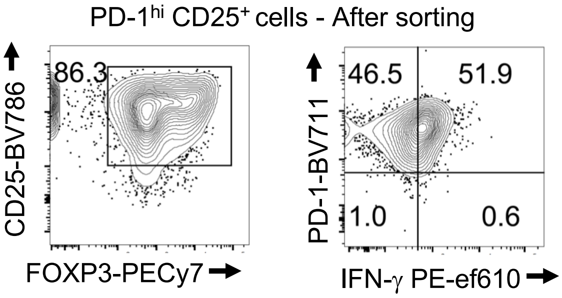

Supplementary Fig. 18. PD-1^{hi}CD25^{+} cell purity. Purified CD4^{+} T cells were activated with TCR stimulation and allowed to expand in the presence of TGF-β1 (10 ng/ml) and IL-2 (100 U/ml). Some cultures were infected with HIV on day 2 after TCR stimulation. PD-1^{hi}CD25^{+} cells were purified from HIV-infected CD4 cultures using sequential sorting of PD-1-PE^{+} cells and CD25^{high} T_{reg} cells using STEMCELL technology PE isolation and CD25+T_{reg} isolation kits. Data represent three independent experiments.

Supplementary Fig. 19

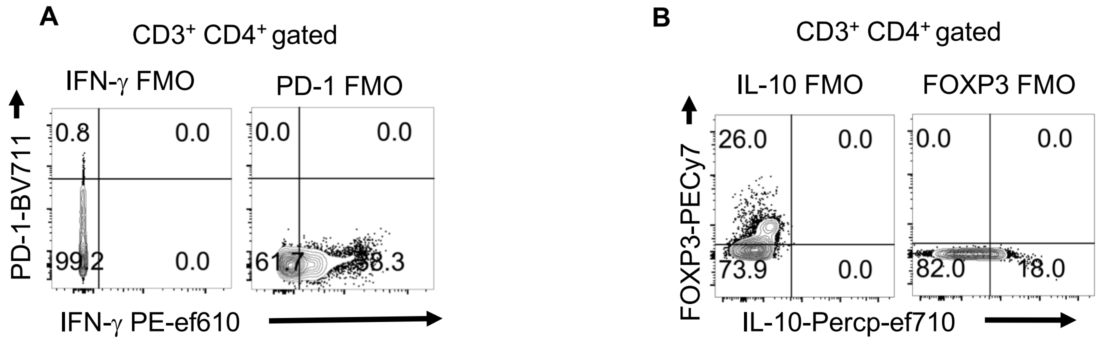

Supplementary Fig. 19. FMO controls to determine the boundary for positively stained cells. PD-1 and IFN-γ (A) and IL-10 and FOXP3 (B) FMO controls in gingival mucosa processed for flow cytometry *ex vivo*. Cells were restimulated with PMA/Ionomycin for 4 hours before flow cytometry. The gates were assigned based on the unstained controls, FMO controls, and non-restimulated control cells for each experiment. Because tissue cells (oral mucosa) may have some autofluorescence, we also performed PBMC staining control cells in parallel. We chose the most appropriate gating based on an objective evaluation using these controls when establishing the staining protocol for experiments. Representative flow cytometric data from 10-12 independent study participants are shown.

Supplementary Table 1. Human participants enrolled in the study

Group	HIV− (n = 32)	HIV+ ART+ (n=46)
Age (years) median	49 +/- 18.5	54 +/- 9.9
Aged 60 and above	21.8%	22.2%
Time under cART median	0	15 +/- 7.6 yrs
Viral load median	0	20 (range 0.8 – 272)
% prior Candidiasis positive	0	34.7%
% periodontitis +	0	4.3%

Source data are provided as a Source Data file.
