# Supplementary material for: Oral immune dysfunction is associated with the expansion of FOXP3+PD-1+Amphiregulin+ T cells during HIV infection
Source: Nat Commun. 2021 Aug 26;12:5143. doi: 10.1038/s41467-021-25340-w (PMC8390677; doi:10.1038/s41467-021-25340-w)
Supplement: Supplementary file 3 — Reporting Summary [file 41467_2021_25340_MOESM3_ESM.pdf]

nature research

Corresponding author(s): Pushpa Pandiyan

Last updated by author(s): May 11, 2021

Reporting Summary

Nature Research wishes to improve the reproducibility of the work that we publish. This form provides structure for consistency and transparency in reporting. For further information on Nature Research policies, see our Editorial Policies and the Editorial Policy Checklist.

Statistics

For all statistical analyses, confirm that the following items are present in the figure legend, table legend, main text, or Methods section.

n/a	Confirmed	
☐	☒	The exact sample size (*n*) for each experimental group/condition, given as a discrete number and unit of measurement
☐	☒	A statement on whether measurements were taken from distinct samples or whether the same sample was measured repeatedly
☐	☒	The statistical test(s) used AND whether they are one- or two-sided<br>*Only common tests should be described solely by name; describe more complex techniques in the Methods section.*
☐	☒	A description of all covariates tested
☐	☒	A description of any assumptions or corrections, such as tests of normality and adjustment for multiple comparisons
☐	☒	A full description of the statistical parameters including central tendency (e.g. means) or other basic estimates (e.g. regression coefficient) AND variation (e.g. standard deviation) or associated estimates of uncertainty (e.g. confidence intervals)
☐	☒	For null hypothesis testing, the test statistic (e.g. *F*, *t*, *r*) with confidence intervals, effect sizes, degrees of freedom and *P* value noted<br>*Give P values as exact values whenever suitable.*
☒	☐	For Bayesian analysis, information on the choice of priors and Markov chain Monte Carlo settings
☒	☐	For hierarchical and complex designs, identification of the appropriate level for tests and full reporting of outcomes
☐	☒	Estimates of effect sizes (e.g. Cohen's *d*, Pearson's *r*), indicating how they were calculated

Our web collection on statistics for biologists contains articles on many of the points above.

Software and code

Policy information about availability of computer code

Data collection	For Flow cytometry: Becton Dickinson Fortessa -BD FACSDiva software ver.7<br>For RNA seq: Illumina Casava1.7 software used for basecaling; HTSeq v0.6.1; STAR v2.5<br>The FASTQ files with 125bp paired-end reads were processed using Trimmomatic (version 0.30) to remove adaptor sequences. The trimmed FASTQ data were aligned to the human genome with STAR (version 2.5), which used GENCODE gtf file version 4 (Ensembl 78). Differential expression analysis: The gene reads count data from HOIL and PBMC samples were normalized with Edge R Package limma (version 3.26.8) and analyzed with an unpaired t-test.
Data analysis	Flowjo versions 9.8, 9.9.6, 10.5.3, and 10.7.1;<br>Graph Pad ver Prism 8;<br>Microsoft Excel 2016, ver 16.16.27;<br>RNA sequencing differential Analysis using Deseq 2,v2_1.6.3, ClusterProfiler v2.4.3, and EdgeR (version 3.26.8); Heatmaps for different cytokine signatures were created in R using the heatmap.2 function in g plots (version 2.17.0).<br>RNA sequencing Enrichment Analysis using GSEA (http://www.broad.mit.edu/GSEA), Molecular Signatures Database v7.4 (http://www.gsea-msigdb.org/gsea/msigdb/index.jsp), Reactome Pathway Database (https://reactome.org), and Gene Ontology enrichment analysis (http://geneontology.org) databases.

For manuscripts utilizing custom algorithms or software that are central to the research but not yet described in published literature, software must be made available to editors and reviewers. We strongly encourage code deposition in a community repository (e.g. GitHub). See the Nature Research guidelines for submitting code & software for further information.

Data

Policy information about availability of data

All manuscripts must include a data availability statement. This statement should provide the following information, where applicable:

- Accession codes, unique identifiers, or web links for publicly available datasets
- A list of figures that have associated raw data
- A description of any restrictions on data availability

RNA sequencing data from healthy human participants that support the findings of this study have been deposited in GEO, NCBI with the GSE167211 accession code. https://www.ncbi.nlm.nih.gov/geo/query/acc.cgi?acc=GSE167211. Transcriptome data from HIV+ patients are deposited at the NCBI Genotypes and Phenotypes (dbGaP)data repository. These data are open to general research use (dbGaP Study Accession: phs002364.v1.p1) and available at https://www.ncbi.nlm.nih.gov/projects/gap/cgi-bin/study.cgi?study_id=phs002364.v1.p1. Other data that support the findings of this study are also available from the corresponding author upon reasonable request.

Field-specific reporting

Please select the one below that is the best fit for your research. If you are not sure, read the appropriate sections before making your selection.

☒ Life sciences ☐ Behavioural & social sciences ☐ Ecological, evolutionary & environmental sciences

For a reference copy of the document with all sections, see nature.com/documents/nr-reporting-summary-flat.pdf

Life sciences study design

All studies must disclose on these points even when the disclosure is negative.

Sample size	Power analyses was done based on estimates. For the power and sample size calculation, we used https://www.stat.ubc.ca/~rollin/stats/ssize/n2.html We used relevant population values for mu1 (mean of population 1), mu2 (mean of population 2), and sigma value of 0.5 (common standard deviation) 0.05 as α (type I error rate) and a 2 sided test for power calculation. Because our preliminary data showed that HIV+ group had atleast 1.4 fold increase in Treg proportions, we used 1 and 1.4 as mu1 and mu2 respectively. Our desired power was set to 0.8.
Data exclusions	Data points were not excluded.
Replication	We performed at least triplicate repeats of the in vitro experiments with independent biological replicates in each experiment. All replicate experiments showed reproducibly similar data and were successful.
Randomization	Human participants were randomly assigned in control and HIV+ groups, with representation of males and females in each group. All other in vitro experiments were also performed using randomly allocated tonsils.
Blinding	ELISAs were performed by a technician who was blinded to the identity of the saliva and supernatant samples. The investigators were also blinded to group allocation during RNA-seq data collection and/or analysis. It was not possible to do complete blinding for the in vitro and flow cytometry experiments, as the same research associate performed the cell culture and the staining. However, the person who did the flow-cytometry data analysis was blinded on the groups until the final analysis of all the replicate experiments at which point the research associate released the codes for the cell-culture groups.

Reporting for specific materials, systems and methods

We require information from authors about some types of materials, experimental systems and methods used in many studies. Here, indicate whether each material, system or method listed is relevant to your study. If you are not sure if a list item applies to your research, read the appropriate section before selecting a response.

Materials & experimental systems

n/a	Involved in the study
☐	☒ Antibodies
☒	☐ Eukaryotic cell lines
☒	☐ Palaeontology and archaeology
☒	☐ Animals and other organisms
☐	☒ Human research participants
☒	☐ Clinical data
☒	☐ Dual use research of concern

Methods

n/a	Involved in the study
☒	☐ ChIP-seq
☐	☒ Flow cytometry
☒	☐ MRI-based neuroimaging

Antibodies

Antibodies used

Information of all the antibodies:
Antibodies Source Catalog number Clone Lot
human CD28(CD28.2) Invitrogen 16-0289-85 CD28.2 2197855
CD25 (M-A251) BD Biosciences 563701 M-A251 9081958
CD4 (OKT4) Invitrogen 56-00480-82 OKT4 (OKT-4) 2114219
CD45 (HI30) Invitrogen 61-0459-1 HI30 2135833
CD8 (RPA-T8) BD Biosciences 561453 RPA-T8 0030878
HLA-DR (LN3) Invitrogen 47-9956-42 LN3 1950154
IFN-γ (4S.B3) Invitrogen 17-7319-82 4S.B3 2193918
IL-17A (eBio64DEC17) Invitrogen 47-7179-42 eBio64Dec17 1952434
FOXP3 (236A/E7) eBioscience 14-4777-82 236A/E7
Phospho-AKT 1 (Ser473) (SDRNR) Invitrogen 48-9715-42 SDRNR 2133291
BCL-6 (BCL-UP) Invitrogen 46-9880-42 BCL-UP 2172654
CXCR5 (MU5UBEE) eBioscience 48-9185-42 MU5UBEE 4275264
Ki-67 (SolA15) Invitrogen 11-5698-82 SolA15 2040334
IL-10 (JES3-9D7) Invitrogen 46-7108-42 JES3-9D7 4331074
AREG (AREG559) Invitrogen 12-5370-42 AREG559 4351598
ST2 (goat polyclonal) Life Technologies corp PA5-47024 Polyclonal
Phospho-caspase 1 (Ser376) Invitrogen PA5-38565 UG2807841
CD279 (PD-1) (EH12.1) BD Biosciences 564017 EH12.1 0087488
CXCR4 (12g5) BD Biosciences 560670 12g5
CCR5 (2D7/CCR5) BD Biosciences 556903 2D7
BCL-2 (Bcl-2/100) BD Biosciences 560637 Bcl-2/100 9268283
CD19 (SJ25C1) BD Biosciences 557835 SJ25C1 6195788
CD38 (HIT2) BD Biosciences 563964 HIT2 7089805
CD3 (HIT3a) BD Biosciences 740073 HIT3a 1049330
IL-1R1 (hIL1R-M1) BD Biosciences 551388 CD121a (IL-1R1) 9297644
Phospho-AEP (SER 226) Millipore sigma ABN2270 Polyclonal
Biotinylated AEP R and D systems BAF2199 Polyclonal
BLIMP1 R and D systems IC36081P 646702
Chimeric PDL-1-Fc R and D systems 156-B7-100 Recombinant
Secondary donkey anti-mouse IgG-BV421 Jackson Immuno Research 715-675-150
Anti-goat IgG (H+L) superclonalTM-Alexa Fluor 647 Invitrogen A27018

Validation

All antibodies were commercially available, validated and were used in previous studies, as per the references available in manufacturer's websites. For the BD- Biosciences flow cytometry antibodies, the company website says "The production process underwent stringent testing and validation to assure that it generates a high-quality conjugate with consistent performance and specific binding activity". The invitrogen/Thermofisher website says: "To help ensure superior antibody results, we've expanded our specificity testing methodology using a 2-part approach for advanced verification".

We also titrated the antibodies using unstained or Isotype controls prior to use.

Human research participants

Policy information about studies involving human research participants

Population characteristics

Informed consents from healthy individuals and Cleveland HIV+ cohort under a protocol approved by the University Hospitals the Cleveland Medical Center Institutional Review Board. n= 78, Males= 49, Females = 29; Healthy control subjects were at least 18 years of age and in good general health (Table.1). HIV+ participants were 18 years or older, and were HIV positive with cART treatment for at least 1 year. > 75 % of HIV+ patients reported prior and current soft tissue lesions, gingivitis, and periodontitis. For the periodontitis study, healthy controls (n=8; 5 females and 3 males) and periodontitis patients (Perio. n= 9; 6 females and 3 males)were recruited under a separate UH-IRB protocol. Demographics was consistent with the general population in the Cleveland-Akron metro area.

Recruitment

Participants were recruited after obtaining informed consents. Healthy control subjects were at least 18 years of age and in good general health. Exclusion criteria were oral inflammatory lesions (including gingivitis and periodontitis), oral cancer diagnosis, soft tissue lesions, and the use of tobacco in the past month. HIV+ participants were 18 years or older, and were HIV positive with cART treatment for at least 1 year. Exclusion criteria were oral cancer diagnosis and the use of tobacco in the past month. The inclusion and exclusion criteria were the same for periodontitis study, except that the the inclusion criteria for the periodontitis group included the presence of periodontitis.

HIV+ individuals volunteered based on their HIV positivity. Studying the HIV+ patients in this Case-Control study was the goal of the investigation and this self-selection did not impact the results negatively.

Ethics oversight

IRB; University Hospitals Cleveland Medical Center Institutional Review Board; IRB# 05-17-02 for the HIV study, and IRB #03-13-15 for the periodontitis study.

Note that full information on the approval of the study protocol must also be provided in the manuscript.

Flow Cytometry

Plots

Confirm that:

☒ The axis labels state the marker and fluorochrome used (e.g. CD4-FITC).

☒ The axis scales are clearly visible. Include numbers along axes only for bottom left plot of group (a 'group' is an analysis of identical markers).

☒ All plots are contour plots with outliers or pseudocolor plots.

☒ A numerical value for number of cells or percentage (with statistics) is provided.

Methodology

Sample preparation	For single-cell flow cytometry staining, cells isolated and processed ex vivo from tissues or lymphoid organs as well as cultured cells were washed in PBS or PBS/BSA, and blocked by Fc receptor blocking, before surface staining using the antibodies. For Foxp3 and other intracellular marker staining, the cells were fixed with Foxp3 fix-perm set (eBioSciences/Thermofisher) after surface staining. Live-Dead viability staining was used to remove dead cells in the analyses. Appropriate un-stain, isotype, secondary antibody, single stain and FMO controls were used and representative data are shown in supplementary figures. Before intracellular cytokine staining, cultures were re-stimulated with PMA (50 ng/ml) and Ionomycin (500 ng/ml) for 4 hours, with brefeldin-A (10 µg/ml) added in last 2 hours. For phospho staining, the cells were washed, fixed and were stained with Phosflow staining kit from BD Biosciences using manufacturer's protocol.
Instrument	BD Fortessa
Software	Flowjo versions 9.8, 9.9.6, 10.5.3, and 10.7.1
Cell population abundance	CD4+ T ells were more abundant in PBMC and tonsils than in the oral tissues.
Gating strategy	Gating strategy: Preliminary FSC/SSC gates for the starting leukocyte cell population, and subsequent gating to include singlets, and CD3+ T cells were used. Boundaries of the "positive" gates were assigned based on the unstained controls, PBMC/d0 negative controls, and FMO controls. We have shown data exemplifying the gating strategies and the controls where appropriate in figures S1B, S1C, S5B, S5D, S8, and S19 in the supplementary Information.

☒ Tick this box to confirm that a figure exemplifying the gating strategy is provided in the Supplementary Information.
